# Bifunctional Small Molecules Enhance Neutrophil Activities Against *Aspergillus fumigatus in vivo* and *in vitro*

**DOI:** 10.3389/fimmu.2019.00644

**Published:** 2019-04-09

**Authors:** Caroline N. Jones, Felix Ellett, Anne L. Robertson, Kevin M. Forrest, Kevin Judice, James M. Balkovec, Martin Springer, James F. Markmann, Jatin M. Vyas, H. Shaw Warren, Daniel Irimia

**Affiliations:** ^1^BioMEMS Resource Center, Department of Surgery, Massachusetts General Hospital, Harvard Medical School, Boston, MA, United States; ^2^Boston Children's Hospital, Harvard Medical School, Boston, MA, United States; ^3^Cidara Therapeutics, San Diego, CA, United States; ^4^Division of Transplantation, Massachusetts General Hospital, Boston, MA, United States; ^5^Division of Infectious Diseases, Massachusetts General Hospital, Harvard Medical School, Boston, MA, United States

**Keywords:** neutrophil, fungi (mycelium and spores), *Aspergillus fumigatus* (*A. fumigatus*), bifunctional molecules, cloudbreak, microfluidic, zebrafish

## Abstract

Aspergillosis is difficult to treat and carries a high mortality rate in immunocompromised patients. Neutrophils play a critical role in control of infection but may be diminished in number and function during immunosuppressive therapies. Here, we measure the effect of three bifunctional small molecules that target *Aspergillus fumigatus* and prime neutrophils to generate a more effective response against the pathogen. The molecules combine two moieties joined by a chemical linker: a targeting moiety (TM) that binds to the surface of the microbial target, and an effector moiety (EM) that interacts with chemoattractant receptors on human neutrophils. We report that the bifunctional compounds enhance the interactions between primary human neutrophils and *A. fumigatus in vitro*, using three microfluidic assay platforms. The bifunctional compounds significantly enhance the recruitment of neutrophils, increase hyphae killing by neutrophils in a uniform concentration of drug, and decrease hyphal tip growth velocity in the presence of neutrophils compared to the antifungal targeting moiety alone. We validated that the bifunctional compounds are also effective *in vivo*, using a zebrafish infection model with neutrophils expressing the appropriate EM receptor. We measured significantly increased phagocytosis of *A. fumigatus* conidia by neutrophils expressing the EM receptor in the presence of the compounds compared to receptor-negative cells. Finally, we demonstrate that treatment with our lead compound significantly improved the antifungal activity of neutrophils from immunosuppressed patients *ex vivo*. This type of bifunctional compounds strategy may be utilized to redirect the immune system to destroy fungal, bacterial, and viral pathogens.

## Introduction

Humans are continuously exposed to airborne spores of the saprophytic fungus *Aspergillus fumigatus* (*A. fumigatus*). In healthy individuals, pulmonary host defense mechanisms efficiently eliminate this mold. However, the incidence of invasive pulmonary aspergillosis (IPA) has risen in recent decades, reflecting the increasing number of immunosuppressive medical interventions such as chemotherapy, hematopoietic stem cell and solid organ transplants (1, 2). Even with appropriate antimicrobial therapy, the mortality rate of IPA remains as high as 50% (3, 4). In a recent clinical study of patients with acute lymphoblastic leukemia (ALL), 6.7% of patients developed invasive fungal infections within a median time of 20 days after induction of chemotherapy, with a high (19.2%) 12-week mortality after diagnosis of invasive aspergillosis (IA) (5). There is an increasing demand for novel therapeutic strategies aimed at enhancing or restoring antifungal immunity (6).

Recently, exploration has begun into the promise of using immunotherapy to combat IA, with use of cytokines and granulocyte transfusions, alone or in combination with antifungal therapy. In the past, chemokines have been tested to modify effector and antigen presenting cells in the context of cancer (7). Modulation of neutrophil functions are an especially promising immunotherapeutic strategy (8). Colony stimulating factors (CSFs) and cytokines, mainly IFN-γ, have been utilized in the clinical management of fungal diseases. CSFs and granulocyte transfusions are used to augment the number and function of circulating neutrophils in neutropenic patients (9). Other *in vivo* studies report on the anti-Aspergillus activity of neutrophils, including the rapid resolution of IPA following recovery of chemotherapy-induced neutropenia (10, 11). *Ex vivo* loading of the antifungal drug posaconazole into HL60s, a neutrophil-like cell line, enhanced activity against *A. fumigatus*, and transfusion of these cells improved survival outcome in a mouse model of IPA (12).

Neutrophils are one of the key targets for fungal immunotherapy because of their critical role in preventing infections. In different immunocompromised murine models, myeloid (notably neutrophils and macrophages), but not lymphoid cells, were strongly recruited to the lungs upon infection. Other myeloid cells, particularly dendritic cells and monocytes, were only recruited to lungs of corticosteroid treated mice, which developed a strong pulmonary inflammation after infection (13). Both macrophages and neutrophils are known to kill conidia, whereas hyphae are killed mainly by neutrophils (14, 15). Some evidence suggests that killing of conidia by neutrophils *in vitro* depends whether or not the conidia are in a “resting” or “swollen” state (16). *In vivo*, early recruitment of neutrophils to the lung is important to inhibit germination of *A. fumigatus* conidia and to restrict growth of hyphae (17). Since hyphae are too large to be engulfed, neutrophils possess an array of extracellular killing mechanisms, including the creation of swarms surrounding the fungi and the formation of neutrophil extracellular traps (NETs), which consist of nuclear DNA decorated with fungicidal proteins (18, 19).

Microfluidics are emerging as an important tool for precisely quantifying neutrophil-pathogen interactions (20). We have recently reported on microfluidic devices that enabled the measurement of neutrophil-fungus interactions at single-cell resolution. We found that human neutrophils have a limited ability to migrate toward and block the growth of *A. fumigatus* conidia (21) and that the growth-blocking ability of human neutrophils is significantly enhanced by peptide chemoattractants such as N-Formyl-Met-Leu-Phe (fMLP), which act through the Formyl Peptide Receptor (FPR1) on neutrophils. This effect of chemoattractants is significantly larger in the presence of chemoattractant gradients compared to uniform concentrations (21). To study interactions between neutrophils and hypha in detail, we have developed an “infection-on-a-chip” device, which enabled the detailed analysis of neutrophil-hypha interaction at single-cell resolution over time and revealed the importance of hypha branching, neutrophil recruitment, and iron sequestration on blocking hypha growth (22).

Here, we present a novel immunotherapy strategy that aims to enhance the interactions between neutrophils and fungi and direct the natural innate immune system to achieve control over fungal infection. Using microfluidic platforms, we quantify a significant increase in recruitment of neutrophils and hyphae killing in both gradients and uniform concentrations of bifunctional compounds that bind both to fungi and neutrophils. We measure decreased hyphal tip growth velocity in the presence of bifunctional compounds compared to the antifungal targeting moiety alone. Using a zebrafish model of conidial phagocytosis, we demonstrate molecular specificity for drug action through human FPR1 *in vivo*. Finally, we demonstrate that these bifunctional compounds significantly improve the antifungal activity of neutrophils from immunosuppressed patients *ex vivo*.

## Results

**Figure 1:**
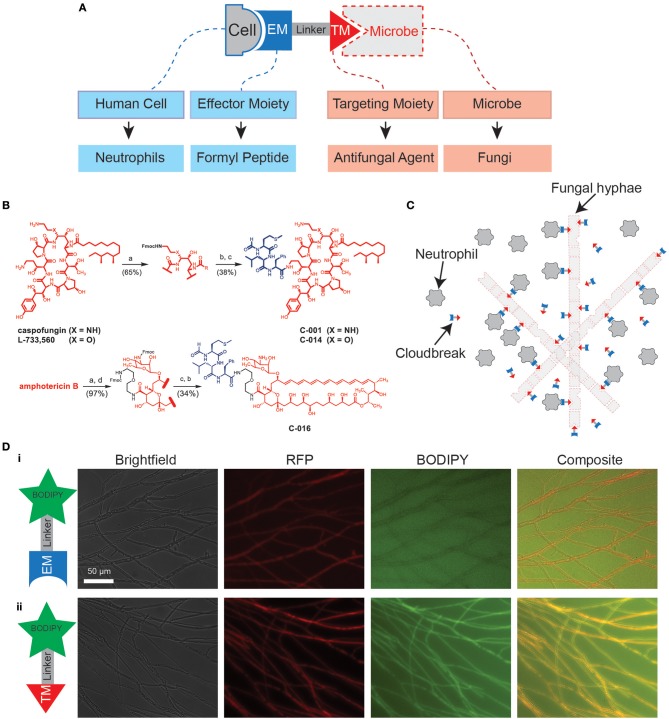
Design of immunogenic bifunctional compounds for enhancing neutrophil activity against fungi. **(A)** Diagram shows conceptual basis of bifunctional compounds design to stimulate interactions between specific immune cell types and microbes. The bifunctional compounds used in this study utilize antifungal TMs and a formyl peptide EM with the aim of stimulating neutrophil activity against fungi. **(B)** Chemical structures detailing synthesis of C-001/C-014 and C-016 by fusion of an fMLP EM with caspofungin (CAS) and amphotericin B (AmB) TM, respectively. Detailed synthesis methods can be found in the Supplementary Material. **(C)** Hypothesized mode of action: Binding of formyl peptides enhances neutrophil activation, while molecules decorating the fungal surface further stimulate their anti-microbial activities. **(D)** Targeting of compounds to the fungal surface via the antifungal TM caspofungin. Following 30 min of treatment with caspofungin-BODIPY conjugate, RFP-expressing hyphal structures are clearly labeled with the fluorescent signal from BODIPY **(ii)**. This specific decoration of the fungal surface does not occur using a BODIPY-conjugated formyl peptide control **(i)**. Scale as shown.

Bifunctional compounds are molecules with two binding sites: a targeting moiety (TM), which recognizes a target on the surface of microbes, and an effector moiety (EM), which binds to a receptor on the surface of the immune cells (7, 23, 24) (Figure 1A). Here, we tested bifunctional compounds that used caspofungin (CAS) and amphotericin B (AmB) as TMs with affinity to known fungal targets: (1-3)-β*-D-glucan synthase* and *ergosterol*, respectively. These compounds were linked to the EM fMLP, which is an FPR1 ligand (Figure 1B, Figure S1). The coupling of the TM to the EM results in bifunctional compounds designed to decorate fungal targets with potent activators of innate immune cells, with the goal of enhancing antifungal activity (Figure 1C). To visualize decoration of fungal hyphae via antifungal TMs, we utilized a boron-dipyrromethane (BODIPY) labeled caspofungin (TM-BODIPY) conjugate. Treatment of RFP-expressing fungal hyphae for 30 min with TM-BODIPY [10 mM] augmented the BODIPY fluorescent signal at the hyphal interface (Figure 1Dii). This effect is most likely due to the specific binding and accumulation of antifungal TM on the surface of the fungi, and was not observed for a BODIPY-labeled formyl peptide (EM-BODIPY) negative control (Figure 1Di).

### Bifunctional Compounds Amplify Human Neutrophil Migration Toward *A. fumigatus* and Suppression of Fungal Growth

To confirm that the bifunctional compounds interact with human neutrophils via FPR1, we tested the ability of the compounds to induce neutrophil chemotaxis. First, we calculated the minimum inhibitory concentrations (MICs) and minimum effective concentration (MEC) of our compounds against *A. fumigatus* (AF293) in the absence of neutrophils (see Supplemental Materials). Compounds C-001 and C-014 (CAS-formyl peptide conjugates), as well as C-016 (a AmB-formyl peptide conjugate) demonstrated potent MIC/MEC values, which suggested excellent affinity of the TMs (Table 1).

**Table 1:** MIC/MEC values (μM) for conjugates and control compounds.

	***A. fumigatus***
**Compound**	**MAY-3626**	**MAY-4609**	**ATCC 13073**
CAS	>29/0.055	>29/0.11	>29/0.11
C-014	>20/0.16	>20/0.076	>20/0.16
AmB	0.54	0.54	0.54
C-016	1.4/1.4	2.8/0.7	2.8/1.4

**Figure 2:**
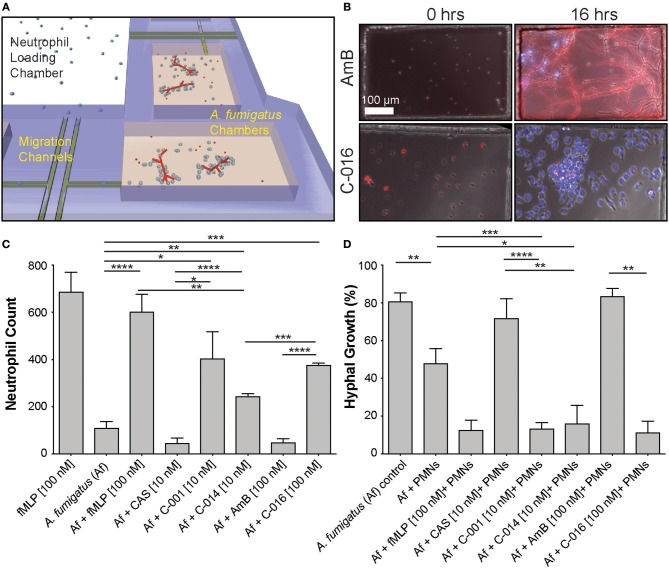
Gradients of bifunctional compounds enhance human neutrophil recruitment and their ability to suppress *A. fumigatus* hyphal growth. **(A)** A previously published device consisting of fungal growth chambers connected via migration channels to one central neutrophil reservoir are used to test neutrophil chemotaxis in response to gradients of bifunctional compounds (21). **(B)** Representative images show *A. fumigatus* (red, RFP) hyphal growth and neutrophil (blue, Hoechst) recruitment in chambers at 0 and 16 h in the presence of C-016 (bifunctional conjugate with amphotericin B TM and formyl peptide EM, lower panels) or amphotericin B (AmB, upper panels) controls. Gradients of C-016 resulted in enhanced neutrophil recruitment and effective suppression of hyphal growth compared to amphotericin B alone. Scale as shown **(C)**. Quantification of neutrophil recruitment at 16 h in response to bifunctional compounds compared to relevant controls. Chemotaxis of neutrophils was enhanced in the presence of the formyl peptide control (fMLP [100 nM]) and all three bifunctional formyl peptide conjugates compared to untreated and antifungal-treated controls. **(D)** Quantification of hyphal growth at 16 h following treatment with bifunctional compounds in the presence of neutrophils compared to relevant controls. Only partial suppression of hyphal growth was observed in the presence of neutrophils alone. This was significantly enhanced by treatment with all three bifunctional conjugates and the formyl peptide control, as previously described (20). Antifungal controls used at the relevant concentrations did not affect fungal growth. Bar graphs show mean and standard error from pooled experimental replicates. Statistics: two-tailed *T*-test. *p ≤ 0.05, **p ≤ 0.01, ***p ≤ 0.001, and ****p < 0.0001.

Next, we validated that these concentrations also induced maximum chemotaxis of human neutrophils. Measurement of healthy-donor neutrophil recruitment showed that C-001, C-014, and C-016 retained potent chemotactic activity. The chemotactic activity of the compounds was comparable to that of an optimal concentration of fMLP (25), with [10 nM] C-001, and [100 nM] of C-016 or fMLP inducing maximum neutrophil migration in the microfluidic assay (Figure 2C). Importantly, CAS and AmB were not chemotactic to neutrophils (Figure 2C).

To investigate the interactions between neutrophils and fungi at single-cell resolution, we utilized our microfluidic infection-on-chip platforms, which provide well-controlled microenvironment conditions (21). In the absence of drug, we observed that low numbers of neutrophils migrate naturally toward *A. fumigatus* hyphae in the chemotaxis-chambers (Figure 2B top panel Movie S1). We tested that human neutrophils are activated in the presence bifunctional compounds by measuring the change in circularity and reactive oxygen species (ROS) production (Figure S2). We also ran a dose-response experiment to identify the optimal concentration of C-001, C-014, and C-016 to induce neutrophil chemotaxis in the presence of *A. fumigatus* (Figures S3, S4). C-001 [10 nM], C-014 [10 nM], and C-016 [100 nM] were able to produce a significant influx of neutrophils compared to *A. fumigatus* alone. The bifunctional compounds were less chemotactic than the fMLP [100 nM] positive control in the presence of *A. fumigatus*, likely due to the lower [10 nM] concentration used for C-001 and C-014 (Figure 2C).

In the chemotaxis-chamber devices, in the absence of neutrophils, 80.7 ± 4.6% of the conidia germinated within 6 h. The antifungal backbone alone had minimal effect on the germination of conidia within the same time interval (CAS: 71.8 ± 10.4% and AmB: 83.4 ± 4.2%). Neutrophils alone reduced the fraction of conidia germination to 47.9 ± 7.8% (*N* = 8). Remarkably, human neutrophils further reduced the fraction of conidia germinating in the presence of C-001 (13.2 ± 3.4, *N* = 10), C-014 (16.0 ± 9.7%, *N* = 4), and C-016 (11.2 ± 6.0%, *N* = 4) (Figure 2D, Movies S2, S3). Maintained conidial fluorescence even following phagocytosis by neutrophils (Figure 2B) indicated that although conidial germination was suppressed, some of these spores likely remained viable within neutrophils over the timeframe imaged.

### Uniform Concentrations of Bifunctional Compounds Significantly Enhance the Activity of Human Neutrophils Against Growing Hyphae

**Figure 3:**
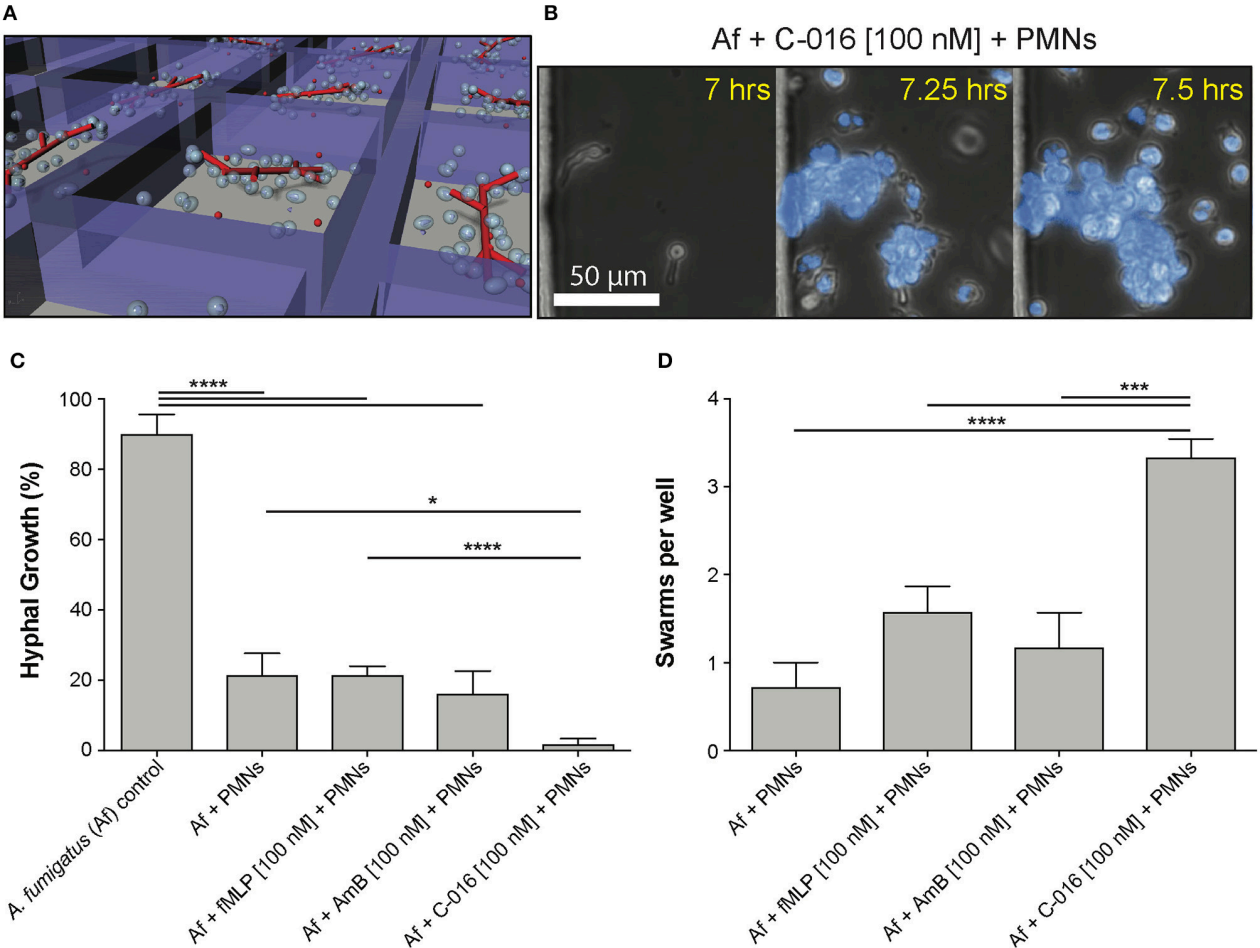
Uniform concentrations of bifunctional compounds enhance the ability of human neutrophils to suppress hyphal growth and stimulate neutrophil swarming. **(A)** Diagram of previously described nanowell device designed to test drug activity at uniform concentrations (21). Fungal conidia are allowed to germinate and grow for 7 h prior to addition of neutrophils in the presence or absence of neutrophils. **(B)** Representative images show swarming of neutrophils (polymorphonucleocytes, PMNs, blue, Hoechst) around *A. fumigatus* hyphae induced by the presence of C-016. **(C)** Quantification of hyphal growth in this device demonstrates significant suppression by neutrophils, either in the presence or absence of fMLP or AmB treatment. This suppression was further enhanced in the presence of C-016. **(D)** Quantification of neutrophil swarms around growing *A. fumigatus* hyphae shows a significant increase in the presence of C-016 vs. control conditions. Bar graphs show mean and standard error from pooled experimental replicates. Statistics: two-tailed *T*-test. *p ≤ 0.05, ***p ≤ 0.001, and ****p < 0.0001.

To measure the interactions between human neutrophils and fungi in uniform concentrations of drug, we confined these interactions within nanowells (300 μm wide × 500 μm long × 50 μm deep) (Figure 3A). We loaded fungi into the wells and allowed the conidia to germinate for 7 h. We added isolated human neutrophils to the wells (average concentration: 30 neutrophils/well), in the presence and absence of uniform concentrations of bifunctional compounds and control chemoattractants, and monitored the interactions between neutrophils and fungi for 18 h. The ability of neutrophils to block conidia germination was enhanced in the presence of C-016 [10 nM – 1.7% conidia germination] compared with uniform concentrations of fMLP [100 nM – 21.4 % conidia germination] (Figure 3C). Strikingly, we also observed a significant increase in the number of neutrophil “swarms” (clusters of more than 6 neutrophils) in the presence of C-016 (Figures 3B,D), which correlated with enhanced suppression of hyphal growth in that condition. This “swarming” effect might have been facilitated by the shorter distances between neutrophils and germinating conidia and faster recruitment of larger neutrophil numbers compared to the chemotaxis-chamber assay.

### Bifunctional Compounds Help Neutrophils Block Hyphal Tip Extension

**Figure 4:**
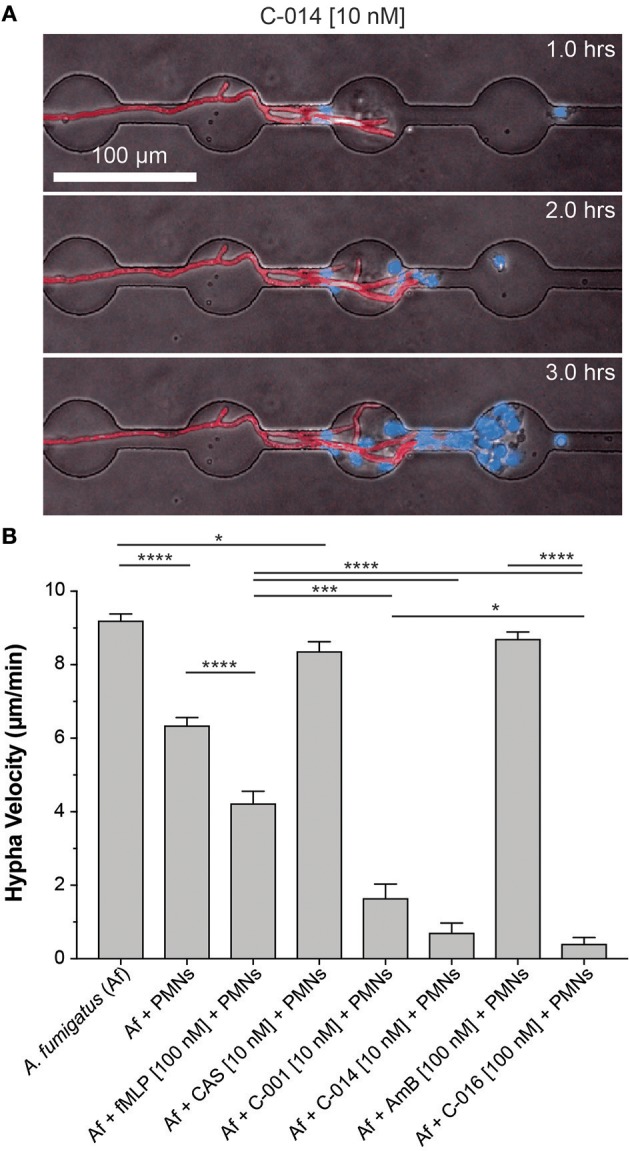
Bifunctional compounds enhance the ability of human neutrophils to suppress hyphal tip extension. **(A)** Representative images show suppression of hyphal tip (red, RFP) elongation by neutrophils (blue, Hoechst) in the presence of C-014 [10 nM]. **(B)** Quantification of hyphal growth velocity demonstrates significant suppression in the presence of bifunctional compounds C-001 [10 nM], C-014 [10 nM], and especially C-016 [100 nM] compared to control conditions—including fMLP [100 nM]. Bar graphs show mean and standard error from pooled experimental replicates. Statistics: two-tailed *T*-test. *p ≤ 0.05, ***p ≤ 0.001, and ****p < 0.0001.

We have previously described the ability of neutrophils to interact with growing hyphal tips and suppress their growth (22). Using similar microfluidic devices (22) that allow fungi to grow for 18 h before interactions with neutrophils and confine growing hyphae into channels, we tested whether bifunctional compounds enhance the interaction between neutrophils and hyphae. We found that the velocity of hypha growth was drastically reduced from ~11 to ~0.5–1.5 μm/min by the presence of human neutrophils and bifunctional compounds (*P* = 0.05, *N* = 10) (Figures 4A,B, Figure S5, Movies S4, S5). The velocity of hypha growth was not altered in the presence of antifungal controls and was only reduced to ~6 μm/min in the presence of neutrophils without the bifunctional compounds.

### Bifunctional Compounds Enhance Phagocytosis of Conidia by Humanized Zebrafish Neutrophils

To assess whether bifunctional compounds could enhance neutrophil responses *in vivo*, we utilized an established zebrafish infection model that has been used to study the activity of innate immune cells in response to *A. fumigatus* conidia and hyphae (26–28). In this model, conidial phagocytosis is heavily predominated by macrophages rather than neutrophils (26, 28). Consequently, reducing macrophage numbers (via knockdown of *spi1* expression using antisense oligonucleotides that block translation of *spi1* mRNA) was required for isolating the effect of neutrophil activities on *A. fumigatus* conidia phagocytosis and clearance (26, 27).

**Figure 5:**
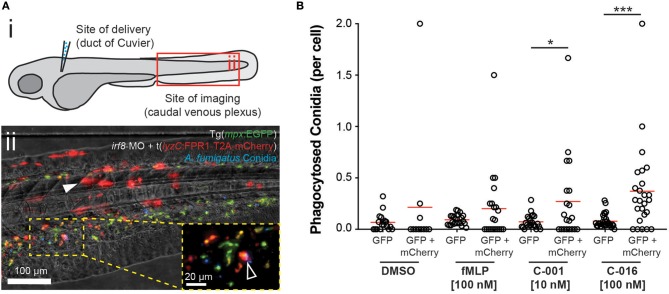
Bifunctional compounds enhance phagocytosis of *A. fumigatus* conidia by zebrafish neutrophils expressing human FPR1. **(A) (i)** Diagram of experimental approach: Calcofluor-stained *A. fumigatus* conidia (blue) are co-delivered with treatments into the circulation via the Duct of Cuvier at 3 days post-fertilization. Imaging is performed at 2 h post-infection at a distal site, the caudal venous plexus, which is rich in leukocytes. **(ii)** Example image of 3 dpf *irf8*-MO treated Tg(*mpx*:EGFP) larva (GFP-labeled neutrophils) with mosaic expression of human FPR1 (traced by mCherry, red fluorescence), 2 h following delivery of calcofluor-labeled (blue fluorescent) *A. fumigatus* conidia. A GFP/mCherry co-labeled leukocyte containing phagocytosed conidia is indicated in magnified panel (open white arrowhead). Off-target expression of the transgene was also observed in tissues including the somites (full white arrowhead). **(B)** Treatment with C-001 and C-016 resulted in significantly increased phagocytosis of conidia by GFP/mCherry+ (human FPR1-expressing) neutrophils compared to GFP(only) wild-type cells. Each point represents an infected larva. *N* ≥ 40 larva scored per condition. Data collated from multiple experiments. Statistics: two-tailed *T*-test. *p ≤ 0.05, ***p ≤ 0.001.

FPR1 sensitivity has been shown to vary widely between mammalian species, with mouse and rat neutrophils exhibiting poor recruitment in response to fMLP compared to human cells (29). There is evidence that zebrafish neutrophils do respond to formylated peptides (30, 31), although experiments in this model have been complicated by inability to distinguish direct responses to chemoattractant from recruitment to injured tissue at the site of microinjection. To avoid this complication in our experiments, we delivered pre-treated conidia at one site (the duct of Cuvier) and analyzed neutrophil responses at a spatially distant site (the caudal venous plexus) (Figure 5Ai).

To test whether bifunctional compounds could enhance phagocytosis of *A. fumigatus* conidia, we microinjected pre-treated and control conidia along with test or control compounds into the circulation, then imaged the caudal venous plexus 2 h post-infection (hpi) (Figure S7A). Despite effective suppression of the macrophage lineage by treatment with antisense oligonucleotides targeting *spi-1* mRNA transcripts (*spi1*-MO) (Figure S7B) and comparable numbers of neutrophils (Figure S7B) and conidia (Figure S7C) present in all groups, no significant increase in the percent of phagocytic neutrophils (Figure S7D) or the number of engulfed conidia (Figure S7E) was observed following pretreatment with bifunctional compounds.

Colony forming units (CFU) provide a poor readout of infectious burden for hyphal pathogens, because unlike single-cell organisms like bacteria or yeast, fungal filaments cannot be reliably homogenized into individual viable units. To assess whether fungal burden might be suppressed following treatment, we therefore scored larvae at 24 hpi for RFP-positive hyphae using fluorescence microscopy (Figure S8). In *spi1*-MO treated larvae, which had neutrophils but reduced macrophages, we observed hyphae in 10–20% of surviving infected larvae, with no significant difference between drug-treated and control groups (Figure S8B). In *spi1-MO/csf3r-MO* treated zebrafish, which had reduced neutrophils as well as macrophages (32), we observed hyphae in 80–90% of larvae, highlighting the important role that neutrophils play in suppressing hyphae in this model. Again, no significant difference was observed between treated and control groups in this context.

Comparison of protein sequence identity between receptor homologs in humans, mice, rats and zebrafish revealed that while the conservation between mammalian homologs was higher than 70%, conservation between mammals and zebrafish was < 40% (Figure S6). To test whether expression of human FPR1 in zebrafish neutrophils could enhance the neutrophil response in the presence of bifunctional compounds, we mosaically expressed human FPR1 under the control of the leukocyte-specific zebrafish *lyzC* promoter (33) using Tol2-mediated transgenesis. Expression of the protein was traced using mCherry linked to the receptor using the self-cleaving T2A peptide, allowing separation of the fluorophore and thus unimpeded receptor function. The transgene DNA construct and Tol2 transposase mRNA were co-injected with an antisense morpholino oligonucleotide targeting *irf8*, knockdown of which results in enhanced specification of neutrophils at the expense of macrophages (34). Injection into Tg(*mpx*:EGFP) embryos at the one-cell stage resulted in both on-target expression of FPR1/mCherry in GFP-labeled neutrophils (GFP/mCherry+ cells), as well as off-target expression in tissues such as the somite (Figure 5Aii). To test whether the FPR1-expressing dual-labeled cells would exhibit an enhanced response to bifunctional compounds, we inoculated control or pre-treated conidia into the circulation FPR1/mCherry-expressing larvae at 3 dpf, and scored phagocytosis at 2 h post-infection in the caudal venous plexus. Because mosaic larvae contained both FPR1-positive (GFP+/mCherry+) and FPR1-negative (GFP(only)+) neutrophils, this approach provided an internal control when assessing phagocytosis of pre-treated conidia.

Pre-treatment of conidia with C-016 prior to inoculation significantly enhanced phagocytosis by GFP+ neutrophils expressing human FPR1 and mCherry (Figure S7E). Furthermore, comparison of per-cell phagocytosis rates demonstrated that pre-treatment of conidia with either C-001 or C-016 (but not DMSO or fMLP) resulted in significantly higher rates of phagocytosis by FPR1/mCherry-expressing GFP+ leukocytes compared to GFP(only)+ cells in the same larvae (Figure 5B). As expected, conidial delivery, leukocyte numbers, and phagocytosis by GFP(only)+ cells (expressing the native zebrafish FPR1) were not significantly different between treatment groups (Figures S7A–D). These observations suggest that using fMLP as an effector moiety on immunotherapy compounds confers species-specific neutrophil responses mediated by differential formyl-peptide receptor activity.

### Bifunctional Compounds Improve Fungicidal Activity of Neutrophils From Immunosuppressed Patients

**Figure 6:**
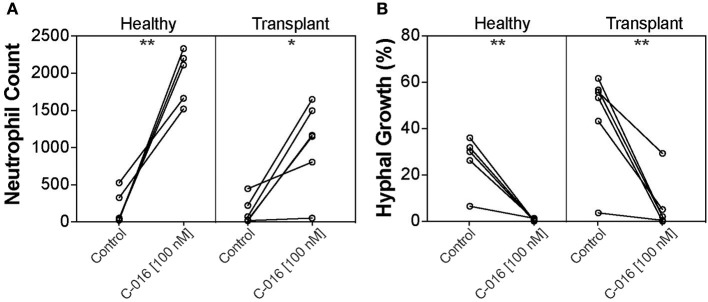
C-016 enhances the anti-fungal activity of neutrophils from immunosuppressed patients. **(A)** Quantification of neutrophil recruitment demonstrated that gradients of C-016 enhanced migration of cells from both healthy donors and transplant patients to Aspergillus chambers compared to unstimulated controls. **(B)** Quantification of hyphal growth showed enhanced suppression by C-016 treated neutrophils from healthy donors and transplant patients compared to unstimulated controls. Each point represents an individual neutrophil donor. *N* = 5 healthy donors and *N* = 6 kidney transplant patient donors tested. Statistics: paired *T*-test. *p ≤ 0.05, **p ≤ 0.01.

Our previous studies have shown that stimulation of neutrophils with chemoattractants presented as spatial gradients, enhanced neutrophil activity against fungal pathogens (20). We therefore assessed the efficacy of C-016, our most promising candidate, in enhancing fungicidal activity of neutrophils isolated from kidney transplant patients using our microfluidic host-pathogen platform. The patients were undergoing various regimes of immunotherapy (Table 2). For healthy donors without stimulation, an average of 194.2 ± 100 neutrophils migrated to the chambers. After stimulation with C-016, an order of magnitude higher number of neutrophils migrated to the chamber (1,966 ± 158.3 cells, *p* = 0.002). For kidney transplant patients without stimulation, an average of 133.5 ± 70.35 neutrophils migrated to the chambers. After stimulation with C-016, an order of magnitude higher number of neutrophils migrated to the chamber (1,053 ± 233.5 cells, *p* = 0.012) (Figure 6A). The increase in migration and stimulation of healthy neutrophils by C-016 resulted in < 1% conidia germination, compared with 26.1 ± 5.1% in the presence of neutrophils without compound. In kidney transplant patients, conidia germination decreased from 45.66 ± 8.8% (neutrophils alone) to 6.47 ± 4.6% (neutrophils and compound) (Figure 6B). One of the transplant patient's neutrophils did not respond to C-016 (Patient #4). In this patient, only 4% of the average number of neutrophils migrated to the chamber, and this was not a sufficient number to control *A. fumigatus* hyphae growth.

**Table 2:** Kidney transplant patient data summary.

**Patient**	**Time from transplant**	**ANC (K/uL)**	**Treatment (daily doses)**	**Neutrophil response to** ***A. fumigatus***			
				**No compound**		**With C-016**	
				**Neutrophils recruited**	**%Fungus alive**	**Neutrophils recruited**	**%Fungus alive**
#1	6 months	12.24	Prograf 4 mg, Prednisone 20 mg, MMF 1 g	222	56.6%	1,648	2%
#2	6 years	1.93	Prograf 4.5 mg, Prednisone 5 mg, MMF 1 g	447	3.6%	806	0.4%
#3	14 years	4.58	Prograf 0.5 mg, Prednisone 2.5 mg, Cell Cept 500 mg	18	61.6%	1166	2%
#4	1 month	0.95	Prograf 6 mg, Prednisone 15 mg, Cell Cept 750 mg, Cefepime, Valcyte and Bactrim	17	55.7%	52	29.3%
#5	7 years	2.47	Prograf 3 mg, Cell Cept 500 mg, Prednisone 5 mg	71	43.2%	1496	5.1%
#6	3 years	9.34	Cyclosporine 125 mg, Cell Cept 500 mg, Prednisone 5 mg	26	53.3%	1,152	0%
Average Transplant Patients				133.5 ± 70.4	45.67 ± 8.8	1,053 ± 233.5	6.47 ± 4.6
Average Healthy Controls				194.2 ± 100	26.1 ± 5.1	1,966 ± 158.3	0.58 ± 0.2
*Comparison between with and without C-0016*						*p =* 0.012	*p =* 0.006

## Discussion

We tested the efficiency of bifunctional compounds consisting of a TM that binds to the surface of *A. fumigatus* and an EM that interacts with FPR1 chemoattractant receptor on human neutrophils in an immunotherapy strategy to amplify neutrophil anti-fungal activities. We found that the bifunctional compounds enhanced the activity of neutrophils against *A. fumigatus* both *in vitro* and *in vivo*. We also measured a significant improvement in the response of human neutrophils isolated from immunosuppressed kidney transplant patients, in *ex vivo* experiments in the presence of bifunctional compound C-016.

We also show that zebrafish models recently developed for the detailed study of leukocyte-fungi interaction during infection (28) are effective tools for evaluating bifunctional compounds *in vivo*. The direct visualization of host-pathogen interactions is facilitated by the use of *Tg(mpx:EGFP/mpeg1:mCherry)* compound transgenic larvae on a *nacre*^−/−^ mutant background with reduced pigmentation (35) to enhance imaging. This compound transgenic line has green fluorescent neutrophils and red fluorescent macrophages (36). Rather than delivering conidia into the zebrafish brain as previously described (26, 27), we instead microinjected fungal conidia directly into the circulation and measured phagocytosis at a spatially distant site. This methodology enabled us to measure neutrophil activity in the absence of damage signals from a nearby wound. Delivery into the circulation resulted in a dominant macrophage phagocytic response, consistent with previous studies (28) and the higher efficiency of macrophages vs. neutrophils at phagocytosing pathogens from zebrafish circulation (37). To allow measurement of neutrophil responses in isolation, macrophage numbers were suppressed by morpholino-mediated knockdown of genes driving macrophage specification from the anterior lateral plate mesoderm (*spi1*) (38), or differentiation from the neutrophil-macrophage common precursor (*irf8*) (34). In theory, the absence of a significant response from zebrafish neutrophils to untreated conidia provided an ideal environment to test enhancement by bifunctional compounds. However, our experiments in zebrafish demonstrate that use of fMLP as an effector moiety provides highly specific activity via the human FPR1.

The modular composition of bifunctional compounds allows for rapid exploration of combinations of TM, EM, and linker domains, potentially enabling efficient discovery of anti-infective molecules with the desired potency, specificity and physical properties. These experiments also highlight the power of utilizing both *ex vivo* and *in vivo* models to test activity, specificity, and mode of action. Together, microfluidics and zebrafish offer complementary imaging-based platforms for measuring leukocyte activity, allowing intuitive translation, and comparison of experimental findings between models.

Bifunctional small molecules represent promising immunotherapies for the treatment of aspergillosis and other fungal infections. Enhancing the host response against fungi is important in situations where the efficacy of the innate immune response is deficient and the degree of the immune suppression in the patient becomes the major host determinant (39). Further study of these agents is warranted. While our current study focusses on enhancing the activity of neutrophils, which express high levels of FPR-1, other cells, such as monocytes, macrophages, dendritic cells, and even vascular endothelial cells and keratinocytes are known to express FPR-1, albeit at lower levels (40). It is possible that activation of these other immune cell lineages *in vivo* may provide further protection against fungi. Treatment with bifunctional compounds may also be limited to topical or localized delivery, for example: to treat dermatophyte infection. Bifunctional compounds may be useful as adjunctive therapy along with standard of care regimens to augment neutrophil killing potential and improve protection against fungal infections. The same compound design principles used here may also be applied to other infectious diseases to redirect the immune system to destroy fungal, bacterial, or viral pathogens. The compendium of microfluidic devices developed to probe neutrophil-fungi interactions (21, 22) could be utilized to prescreen drug candidates and predict the effectiveness of bifunctional compound immunotherapies in individual patients. Theoretically, this type of measurement could also be used to tune the immune system by immunosuppressive therapy drug dosages high enough to avoid organ rejection and low enough to ward off fungal infections.

## Materials and Methods

### Bifunctional Compound Synthesis

Compounds were prepared as described in detail in the (Figure S1). Preparation of C-001: mono-Fmoc-protected caspofungin was prepared from commercial caspofungin acetate by treating with 9-fluorenylmethyl-N-hydroxysuccinimidyl carbonate (Fmoc-OSu) in DMF. The purified product was coupled with N-formyl-L-methioninyl-L-leucyl-L-phenylalanine N-hydroxysuccinimide ester (fMLF-OSu). The Fmoc group was removed from that product by stirring with 10% piperidine to give C-001 after HPLC purification. Preparation of C-014: C-014 was prepared using a procedure analogous to that for C-001 above but replacing caspofungin with L-733,560 (41). Preparation of C-016: The diaminoethylether amide of amphotericin B was prepared by Fmoc derivatization of the mycosamine of amphotericin B followed by coupling with N-Fmoc-diaminoaminoethyl amine and removal of the Fmoc groups with piperidine. Treatment of the product with fMLF-OSu gave C-016 after reversed phase purification. fMet-Leu-Phe (fMLP) was obtained commercially.

### Fungal Strains

*Aspergillus fumigatus* strain 293 expressing cytosolic RFP or GFP was grown on Sabouraud dextrose agar plates supplemented with 100 μg/mL ampicillin at 30°C for 3–4 days. Conidia were harvested by gentle scraping, followed by washing in ice-cold phosphate-buffered saline (PBS) 3 times. Conidia were immediately used or stored at 4°C for up to 1 week before use. To enable visualization following zebrafish infection, conidia were briefly stained with Calcofluor White as previously described (28).

### Zebrafish

Zebrafish stocks were maintained and mated according to standard protocols (42) and following the rules of the Massachusetts General Hospital Subcommittee on Research Animal Care. Transgenic strains—*Tg(mpx:GFPuwm1)* (43) and *Tg(mpeg1:mCherry)* (36), were on the *nacre*^−/−^ background (35), and were a kind gift from Elliott Hagedorn and Leonard Zon. Human formyl-peptide receptor (FPR1) was sub-cloned from pBGSA FPR1-EGFP (Addgene ID:62604) into a middle entry vector and combined with existing 5′ (lyzC promoter) and 3′ (T2A-mCherry) vectors using standard Gateway approaches. Mosaic expression of FPR1 was achieved by Tol2 transposase-mediated transgenesis (44). Briefly, fertilized eggs were co-injected with transgene DNA (50 ng/μl) and Tol2 transposase mRNA (25 ng/μL) into the cell at the single-cell stage. For infection, embryos were raised to 52 h post-fertilization, conidia delivered into the duct of Cuvier by microinjection as previously described (28), and imaging performed on the caudal venous plexus 2 h post-infection to assess phagocytosis. For knockdown studies, fertilized eggs were microinjected with 1 nL of morpholino at the one-cell stage. To enable better measurement of neutrophil-specific responses, primitive macrophage differentiation was restricted by blocking translation of *spi1* or *irf8* using anti-sense morpholino oligonucleotides (*spi1*-MO) as previously described (34, 38). The morphant larvae were raised to 2 days post-fertilization, and then microinjected into the vasculature with a solution of *A. fumigatus* conidia pre-stained with Calcofluor together with C-001 (10 nM), C-016 (100 nM), or DMSO, using microstructured surface arrays developed for this purpose (31, 45). Imaging was performed on a fully automated Nikon TiE microscope. For each larva, a 21-slice z-stack (100 μm at 5 μm intervals) was captured of the caudal venous plexus at 10x magnification for 4 channels: DAPI—conidia, GFP—neutrophils, TRITC—macrophages, and brightfield. Analysis was performed manually using NIS Elements and ImageJ.

### Microfluidic Device Fabrication

Microfluidic devices used to measure leukocyte migration in response to *Aspergillus fumigatus* with or without drug (C-001, C-014, C-016), anti-fungal control (Caspofungin) and/or chemoattractant (fMLP) gradients were manufactured using standard microfabrication techniques. Two layers of photoresist (SU8; Microchem), the first one 10 μm thin (corresponding to the migration channels) and the second one 70 μm thick (corresponding to the FCCs) were patterned on one silicon wafer sequentially using two photolithographic masks and processing cycles according to the instructions from the manufacturer. The wafer with patterned photoresist was used as a mold to produce polydimethylsiloxane (PDMS) (Fisher Scientific) devices, which were then bonded to the base of glass-bottom 12- or 24-well plates, using an oxygen plasma machine (Nordson-March).

### Primary Human Neutrophil Isolation

Peripheral blood samples were collected in 3 mL tubes containing a final concentration of 5 mM ethylenediaminetetraacetic acid (EDTA, Vacutainer; Becton Dickinson) and processed within 2 h of collection.

Using standard sterile techniques, we isolated neutrophils from whole blood by use of HetaSep followed by the EasySep human neutrophil enrichment kit (Stemcell Technologies) in accordance with the manufacturer's protocol. The purity of neutrophils was assessed to be >98%, using the Sysmex KX-21N Hematology Analyzer (Sysmex America). White blood cells (WBCs) were isolated using Hetasep, followed by a 5-min spin-down cycle and washing with 1 × PBS. WBCs were stained with Hoechst fluorescent dye (32.4 μM; Sigma-Aldrich). The final aliquots of WBCs were re-suspended in Roswell Park Memorial Institute (RPMI) medium plus 10% fetal bovine serum (FBS; stock 50 mL of FBS/450 mL of RPMI; Sigma-Aldrich) at a concentration of 4,000 cells/2 μL and kept at 37°C. Cells were then immediately introduced into the microfluidic device for the chemotaxis and A. fumigatus assay. All experiments were repeated at least 3 times with neutrophils or WBCs from 3 different healthy donors.

### Microfluidic Neutrophil Chemotaxis and *A. fumigatus* Killing Assay Preparation

Immediately after bonding to the well plate, donut-shaped devices were filled with *A. fumigatus* conidia (MYA-4609) expressing red fluorescent protein (RFP) at a concentration of 10^6^ cells/mL^+/−^ drug [10 nM], anti-fungal control [10 nM] and/or chemoattractant solution of fMLP [100 nM] (Sigma-Aldrich, St. Louis, MO) in IMDM + 20% FBS. The device was then placed in a vacuum for 15 min. The chemoattractant filled all of the FCCs as the air was displaced. The devices were then vigorously washed five times with 1 × PBS to remove any residual *A. fumigatus* conidia, K2 Therapeutics drug or chemoattractant that was outside of the focal chemotaxis chambers (FCCs). The device was then submerged in 0.5 mL of cell media. Neutrophils or white blood cells (20,000 cells/2 μL) were then pipetted into the cell loading chamber (CLC) using a gel-loading pipette tip and could reach the fungus only after migrating through a 600 μm long channel between the cell-loading well and the drug-treated fungi chambers (Figure 2A). Neutrophil migration into the migration channel toward the FCC started immediately and was recorded using time-lapse imaging for 18 h on a fully automated Nikon TiE microscope (10 × magnification) with biochamber heated to 37°C with 5% carbon dioxide gas. Image analysis of cell migration counts and fungal growth was analyzed by hand using Image J software.

### Statistical Analysis of Neutrophil Chemotaxis and *A. fumigatus* Killing

Image analysis of cell migration counts and fungi growth was analyzed by hand using Image J software. Neutrophils in each chamber were counted every 15 min for the first 2 h of the experiment and then every hour for the remaining 16 h. Percentage of conidia to convert to hyphal growth was measured by counting conidia loaded per chamber before neutrophils or WBCs are loaded into the chamber and counting numbers of these conidia that grow hyphae by 18 h. Fungal growth velocity was calculated using Image J. For experiments with neutrophils from transplant patients, the 16 chambers in each device (*n* = 3) were analyzed for at least three different healthy donors. Data was analyzed for statistical significance using paired two-tailed *t*-tests. For zebrafish experiments, data was tested for normality using the D'Agostino & Pearson normality test. Normally distributed data was analyzed using two-tailed unpaired *t*-tests for pairwise comparisons, or ordinary one-way ANOVA with Tukey's multiple comparisons test. Non-normal data was compared using Kruskal-Wallis test for multiple comparisons. All statistical analysis was performed using Prism Version 7.0a (GraphPad).

## Ethics Statement

De-identified fresh blood samples (volume, 10 mL) obtained from healthy volunteers aged ≥18 years who were not receiving immunosuppressant agents were purchased from Research Blood Components. Blood samples were collected from kidney transplant recipients at Massachusetts General Hospital (MGH). Venous blood samples from healthy volunteers were collected by phlebotomy, after receipt of written informed consent, and the procedures described below were approved by the MGH Institutional Review Board (protocol 2008-P-002123). Zebrafish used in this study were inoculated at 2 days post-fertilization, scored over the following 24 h, and sacrificed at the end of the third day. Microinjection of larvae was approved by the Massachusetts General Hospital Subcommittee on Research Animal Care under Protocol 2011N000127. This protocol adheres to the federal Health Research Extension Act and the Public Health Service Policy on the Humane Care and Use of Laboratory Animals, overseen by the National Institutes of Health (NIH) Office of Laboratory Animal Welfare (OLAW).

## Author Contributions

CJ and FE performed experiments. AR, KF, KJ, JB, MS, JM, JV, and HW provided reagents and oversight. DI provided direct supervision of the work.

### Conflict of Interest Statement

KF, KJ, and HW were cofounders of Cidara Therapeutics. KF, KJ, HW, and JB have filed for patent protection of Cloudbreak compounds. The remaining authors declare that the research was conducted in the absence of any commercial or financial relationships that could be construed as a potential conflict of interest.
