# Supplementary material for: Bifunctional Small Molecules Enhance Neutrophil Activities Against Aspergillus fumigatus in vivo and in vitro
Source: Front Immunol. 2019 Apr 9;10:644. doi: 10.3389/fimmu.2019.00644 (PMC6465576; doi:10.3389/fimmu.2019.00644)
Supplement: Supplementary file 6 [file Data_Sheet_1.PDF]

Supplementary Material

Bifunctional small molecules enhance neutrophil activities against Aspergillus fumigatus in vivo and in vitro

Caroline N. Jones[1,3,7*], Felix Ellett[1,3*], Anne L. Robertson[5], Kevin M. Forrest[6], Kevin Judice[6], James M. Balkovec[6], Martin Springer[6], James F. Markmann[2,3], Jatin M. Vyas[4], Shaw H. Warren[4] and Daniel Irimia[1,3]

[1]BioMEMS Resource Center, [2]Division of Transplantation, [3]Department of Surgery, [4]Division of Infectious Diseases, Massachusetts General Hospital and Harvard Medical School, Boston, [5]Boston Children's Hospital and Harvard Medical School, Boston, [6]Cidara Therapeutics, San Diego, California, [7]Current address: Department of Biological Sciences, Virginia Tech, *Contributed equally

Supplemental Methods

- Synthesis of Bifunctional Compounds
- Minimal Inhibitory Concentration (MIC) Assay Procedure

Supplemental Figures

- Figure S1. Synthesis of Bifunctional Compounds (Cloudbreak).
- Figure S2. Human neutrophils are activated following treatment with bifunctional compounds
- Figure S3. Dose-response curve showing neutrophil recruitment *in vitro*
- Figure S4. Representative images of chemotaxis chambers containing *A. fumigatus* in the presence of C-001 and C-014
- Figure S5. Representative images of neutrophil recruitment towards growing hyphae in the presence of C-014 and CAS control.
- Figure S6. Divergence of formyl-peptide receptor (FPR-1) identity between mammals and zebrafish
- Figure S7. Cell counts and raw phagocytosis scores for zebrafish with mosaic leukocyte expression of human FPR-1.
- Figure S8. Bifunctional compounds do not enhance phagocytosis of conidia by wild-type zebrafish neutrophils.
- Figure S9: Treatment with bifunctional compounds does not enhance the neutrophil-dependent suppression of fungal hyphae in wild-type zebrafish.

Supplemental Movies

- Movie S1: Neutrophils interacting with *A. fumigatus* in chemotaxis chamber device.
- Movie S2: Neutrophils interacting with *A. fumigatus* in chemotaxis chamber device + C-001.
- Movie S3: Neutrophils interacting with *A. fumigatus* in chemotaxis chamber device + C-016
- Movie S4: Neutrophils interacting with *A. fumigatus* hyphae in microfluidic device.
- Movie S5: Neutrophils interacting with *A. fumigatus* hyphae in microfluidic device + C-016.

Supplementary Methods

Synthesis of Bifunctional Compounds

Preparation of C-001

Step a. Preparation of Fmoc-Caspofungin.

9-Fluorenylmethyl-N-hydroxysuccinimidyl carbonate(Fmoc-OSu) (262 mg, 0.78 mmol, in 3 mL of DMF) was added, dropwise, to a stirring solution of caspofungin acetate (860 mg, 0.71 mmol) in DMF (12 mL), cooled to -45°C (acetonitrile dry ice bath) under an atmosphere of nitrogen. The reaction was stirred for 90 minutes while gradually rising to room temperature at which point both mono and bis FMOC adducts were observed by LC/MS. The solvent was reduced to approximately 6 mL on the rotary evaporator and applied directly to reversed phase HPLC purification (Isco CombiFlash Rf; 50g Redisep C18 column; 20 to 95% acetonitrile in DI water containing 0.1% trifluoroacetic acid acid: 15 minute gradient). The pure fractions were pooled and concentrated on the rotary evaporator to afford 660 mg of the product as a white solid (trifluoroacetate salt). Yield: 65%. LC/MS, [M/2+H]+ 658.4, 658.3 calculated.

Step b. Conjugation of Fmoc-Caspofungin with fMLF

To a stirring solution of the mono-FMOC-protected-caspofungin trifluoroacetate (1.23g, 0.87 mmol) dissolved in DMF (8 mL) was added N-methyl morpholine (296 µL, 3 mmol) and formyl-Met-Leu-Phe N-hydroxysuccinimide ester (fMLF-OSu) (534 mg, 1.00 mmol, in 4 mL DMF) in one aliquot. The reaction was stirred for 12 hours and then subjected directly to reversed phase HPLC purification (Isco CombiFlash Rf; 50 g Redisep C18 column; 20 to 95% acetonitrile in DI water containing 0.1% trifluoroacetic acid: 15 minute gradient). The pure fractions were combined and concentrated on the rotary evaporator to afford 975 mg of the product as a white solid. LC/MS, [M/2+H]+ 867.9, 867.5 calculated.

Step c. Deprotection of Fmoc-Protected fMLF-Caspofungin to Give C-001

To a stirring solution of Fmoc-protected-fMLF-caspofungin (975 mg, 0.56 mmol, in 3 mL of DMF) was added 10% piperidine solution in DMF (3 mL) . The reaction was stirred at ambient temperature for 45 minutes at which point complete conversion was observed by LC/MS analysis. The mixture was applied directly to reversed phase HPLC purification (Isco CombiFlash Rf; 50g Redisep C18 column; 5 to 95% acetonitrile in DI water containing 0.1% formic acid: 15 minute gradient). The pure fractions were pooled and the bulk of the acetonitrile was removed on a rotary evaporator and lyophilized to afford 535 mg of product as a white solid, formate salt. HPLC (UV at 220 nm), TR 6.962 min (92.9% purity); 1H-NMR (400 MHz, Methanol-d4) δ 8.10 (s, 1H), 7.18-7.29 (m, 5H), 7.13-7.16 (m, 2H), 6.74-6.78 (m, 2H), 5.00 (m, 1H), 4.90 (d, 1H, J = 2.5 Hz), 4.48-4.61 (m, 6H), 4.26-4.36 (m, 4H), 4.18-4.22 (m, 2H), 3.97-4.01 (m, 2H), 3.94-3.97 (m, 1H), 3.77-3.87 (m, 3H), 3.11-3.28 (m, 3H), 2.79-3.01 (m, 3H), 2.42-2.60 (m, 2H), 2.19-2.30 (m, 3H), 2.10 (s, 3H), 1.90-2.12 (m, 4H), 1.21-1.83 (m, 25H), 1.04-1.14 (m, 2H), 0.84-0.96 (m, 16H); LC/MS, [M/2+H]+ 757.0, 757.4 calculated.

<u>Preparation of C-014</u>

In a procedure analogous to that used for the preparation of C-001, C-014 was prepared starting with L-733,560, an echinocandin analog similar in structure to caspofungin, which also binds to flks1 and inhibits β-1,3-glucan synthase in fungi. The final product had the following physical and spectral characteristics: HPLC (UV at 220 nm), TR 7.812 min (100%); H-NMR (400 MHz, Methanol-d4) δ 8. 09 (s, 1H), 7.20-7.29 (m, 5H), 7.14-7.16 (m, 2H), 6.74-6.77 (m, 2H), 5.18 (d, 1H, J = 3.9 Hz), 4.98 (d, 1H, J = 2.9 Hz), 4.90-4.91 (m, 1 H), 4.44-4.63 (m, 7H), 4.22-4.34 (m, 5H), 3.92-4.01 (m, 2H), 3.69-3.83 (m, 3H), 3.60-3.66 (m, 1H), 3.51-3.56 (m, 1H), 3.33-3.40 (m, 1H), 3.14-3.22 (m, 2H), 2.95-3.07 (m, 3H), 2.41-2.58 (m, 2H), 2.22-2.30 (m, 2H), 2.22-2.30 (m, 3H), 2.10 (s, 3H), 1.90

(s, 3H), 1.67-2.10 (m, 6H), 1.17-1.67 (m, 22H), 1.05-1.13 (m, 2H), 0.85-0.95 (m, 16H); LC/MS, [M+H]+ 1513.7, 1513.8 calculated.

Preparation of C-016

Step a. Preparation of fMLF-OSu

A solution of (N-formyl)-L-methioninyl-L-leucyl-L-phenylalanine (0.866 g, 1.49 mmol) in DMF (65 mL) was charged with N-hydroxysuccinimide (0.171 g, 1.49 mmol) and DCC (0.322 g, 1.56 mmol), and allowed to stir 48 h at room temperature, during which additional N-hydroxysuccinimide (0.034 g, 0.30 mmol) and DCC (0.064 g, 0.31 mmol) were added at t = 24 h. The mixture was used in subsequent steps directly without prior isolation of fMLF-OSu. MS (M+H) 535.0. Alternatively, the crude fMLF-OSu could be isolated by removal of the solvent under reduced pressure.

Step b. Synthesis of N-Fmoc-Mycosamine-Amphotericin B

A solution of amphotericin B (5.00 g, 5.41 mmol) and Fmoc-OSu (2.01 g, 5.95 mmol) in DMF (100 mL) stirred at room temperature for 19 h. The reaction was then diluted with t-butyl methyl ether (1.0 L), and the resulting mixture was filtered through a fritted funnel. The filter cake was then washed with additional t-butyl methyl ether (2 x 100 mL), dried under vacuum, and not purified further. Yield: 5.77 g as a yellow solid. 1H NMR (400 MHz, DMSO-d6) d ppm 0.91 (d, J=7.08 Hz, 3 H) 1.04 (d, J=6.35 Hz, 3 H) 1.09 - 1.13 (m, 4 H) 1.17 (d, J=5.27 Hz, 3 H) 6.92 (d, J=8.44 Hz, 1 H) 7.28 - 7.38 (m, 2 H) 7.38 - 7.48 (m, 2 H) 7.76 (dd, J=7.47, 3.76 Hz, 2 H) 7.89 (d, J=7.52 Hz, 2 H). MS (M+Na+)+ 1169.4.

Step c. Synthesis of N-Fmoc-Mycosamine-Amphotericin B-N-Fmoc-2-(2-aminoethoxy)-ethyl-amide

A solution of N-Fmoc-mycosamine-amphotericin B (4.56 g, 3.98 mmol) in DMF (90 mL) was chilled to ~0°C in an ice/water bath, then charged with

H2NCH2CH2OCH2CH2NHFmoc-HCl (1.59 g, 4.38 mmol), COMU (1.87 g, 4.38 mmol), and DIPEA (1.54 g, 11.9 mmol). After warming to rt and stirring for 2 h, the reaction was diluted with t-butyl methyl ether (900 mL), and the resulting mixture was filtered through a fritted funnel. The filter cake was then washed with additional t-butyl methyl ether (2 x 100 mL), dried under vacuum, and not purified further. Yield: 6.05 g of a yellow solid. 1H NMR (400 MHz, DMSO-d6) d ppm 0.92 (d, J=6.98 Hz, 3 H) 1.04 (d, J=6.35 Hz, 3 H) 1.09 - 1.13 (m, 4 H) 1.16 (d, J=4.49 Hz, 3 H) 6.86 - 6.99 (m, 1 H) 7.20 - 7.26 (m, 1 H) 7.28 - 7.36 (m, 4 H) 7.37 - 7.46 (m, 4 H) 7.61 (d, J=7.52 Hz, 1 H) 7.71 - 7.79 (m, 4 H) 7.81 - 7.92 (m, 4 H). No definitive molecular ion observed by MS.

Step d. Removal of Fmoc Groups to Yield Amphotericin B (2-aminoethoxy)-ethyl-amide

A solution of the N-Fmoc-mycosamine-amphotericin-N-Fmoc-2-(2-aminoethoxy)-ethyl-amide from step c) above (6.05 g, th. 5.79 g, 3.98 mmol) in DMF (30 mL) was charged with piperidine (1.02 g, 11.9 mmol). After stirring at rt for 1.25 h, the reaction was diluted with t-butyl methyl ether (300 mL), and the resulting mixture was filtered through a fritted funnel. The filter cake was then washed with additional t-butyl methyl ether (2 x 100 mL), dried under vacuum, and not purified further. Yield of product: 4.12 g (>100% (102%)) as a yellow solid. 1H NMR (400 MHz, DMSO-d6) d ppm 0.91 (d, J=7.03 Hz, 3 H) 1.04 (d, J=6.30 Hz, 3 H) 1.08 - 1.13 (m, 4 H) 1.14 (d, J=5.91 Hz, 3 H), 8.00 - 8.07 (m, 1 H). MS (M+H+)+ 1010.4.

Step e. Preparation of C-016

fMLF-OSu from Step a) (~1.5 mmol) was charged with amphotericin B (2-aminoethoxy)-ethyl-amide from Step d) (2.00 g, 1.98 mmol). After stirring at room temperature for 1.75 h, the reaction was diluted with t-butyl methyl ether (650 mL), and the resulting mixture was filtered through a fritted funnel. The filter cake was then washed with additional t-butyl methyl ether (2 x 100 mL) and dried under vacuum. The crude product was then mixed with celite (10.8 g) and chromatographed on a 50 g Isco

Gold RediSep C-18 reverse phase silica cartridge, eluting with a gradient from 99.9:0.1 H2O:HOAc to 99.9:0.1 MeOH:HOAc. The isolated product was then lyophilized from H2O (~8 mL). Yield: 0.532 g as a yellow solid. 1H NMR (400 MHz, DMSO-d6) d ppm 0.80 (d, J=6.44 Hz, 3 H) 0.85 (d, J=6.54 Hz, 3 H) 0.91 (d, J=7.03 Hz, 3 H) 1.04 (d, J=6.49 Hz, 3 H) 1.07 - 1.13 (m, 4 H) 1.15 (d, J=5.86 Hz, 3 H) 2.01 (s, 3 H) 7.08 - 7.28 (m, 5 H) 7.89 (d, J=7.91 Hz, 1 H) 7.95 - 8.00 (m, 2 H) 8.01 (s, 1 H) 8.08 (d, J=8.00 Hz, 1 H) 8.27 - 8.35 (m, 1 H). MS (M+H) 1430.2. Analytical HPLC tR = 7.265 min; purity = 100% by ELSD detection.

Minimal Inhibitory Concentration (MIC) Assay Procedure

The MIC assay method essentially followed the procedure described by CLSI (1) and employed automated liquid handlers to conduct serial dilutions and liquid transfers. Automated liquid handlers included the Multidrop 384 (Labsystems, Helsinki, Finland), Biomek 2000 and Biomek FX (Beckman Coulter, Fullerton CA). The wells in columns 2-12 in standard 96-well microdilution plates (Costar 3795) were filled with 150 µl of the correct diluent. These would become the 'mother plates' from which 'daughter' or test plates would be prepared. The drugs (300 µL at 40X the desired top concentration in the test plates) were dispensed into the appropriate well in Column 1 of the mother plates. The Biomek 2000 was used to make serial 2-fold dilutions through Column 11 in the "mother plate". The wells of Column 12 contained no drug and served as the organism growth control wells.

The daughter plates were loaded with 185 µL per well of RPMI described above using the Multidrop 384. The daughter plates were prepared using the Biomek FX which

transferred 5 µL of drug solution from each well of a mother plate to the corresponding well of the daughter plate in a single step.

A standardized inoculum of each organism was prepared per CLSI methods (1). For the Aspergillus isolates, previously prepared and quantitated suspensions were used to make dilutions in RPMI to reach 20X of the final concentration. These dilutions were also transferred to compartments of sterile reservoirs divided by length (Beckman Coulter). The final concentration of the Aspergillus isolates was 0.2-2.5 x 104 CFU/mL.

The Biomek 2000 was used to inoculate the plates. Daughter plates were placed on the Biomek 2000 work surface reversed so that inoculation took place from low to high drug concentration. The Biomek 2000 delivered 10 µL of standardized inoculum into each well. Thus, the wells of the daughter plates ultimately contained 185 µL of RPMI, 5 µL of drug solution, and 10 µL of inoculum. The final concentration of DMSO (if used as a solvent) in the test well was 2.5%.

Plates were stacked 3 high, covered with a lid on the top plate, placed into plastic bags, and incubated at 35ºC for approximately 24-48 hr prior to reading. Plates were read when inoculum was confluent in growth wells or when suggested by CLSI. Plates were viewed from the bottom using a plate viewer. An un-inoculated solubility control plate was observed for evidence of drug precipitation. MICs were read where visible growth of the organism was inhibited. MECs were read where the growth shifted to a small, rounded, compact hyphal form as compared to the hyphal growth seen in the growth control well.

Reference

1.) Clinical and Laboratory Standards Institute (CLSI). Reference Method for Broth Dilution Antifungal Susceptibility Testing of Filamentous Fungi; Approved Standard—Second Edition. CLSI document M38-A2 [ISBN 1-56238-668-9]. CLSI, 940 West Valley Road, Suite 1400, Wayne, Pennsylvania 19087-1898 USA, 2008.

Supplementary Figures

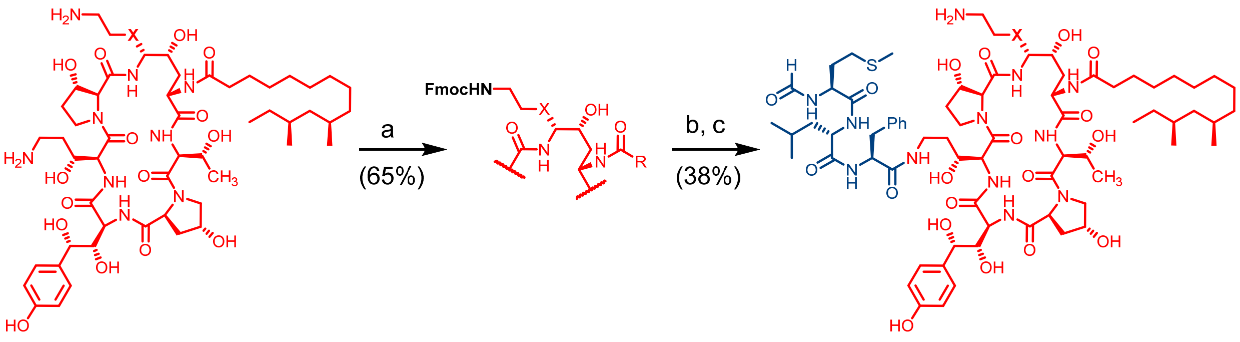

caspofungin (X = NH)
L-733,560 (X = O)

C-001 (X = NH)
C-014 (X = O)

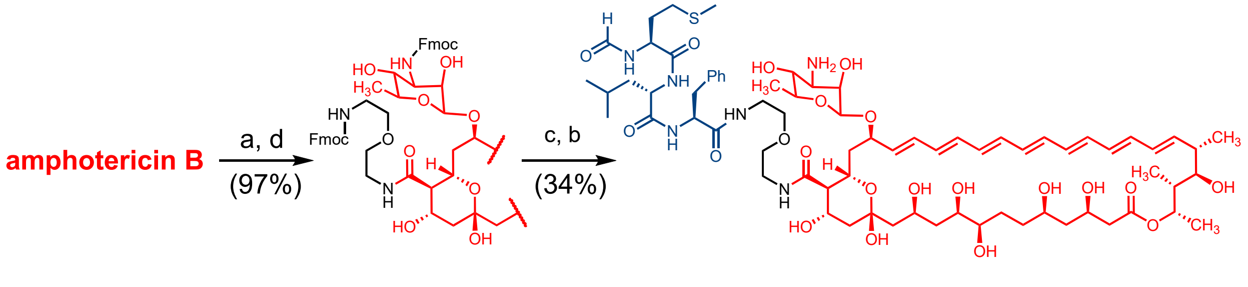

C-016

Figure S1. Synthesis of Bifunctional Compounds (Cloudbreak). a) Fmoc-OSu; b) fMLF-OSu; c) piperidine; d) Fmoc-NH-$(CH_2)_2$-O-$(CH_2)_2$-NH_2, COMU, DIPEA

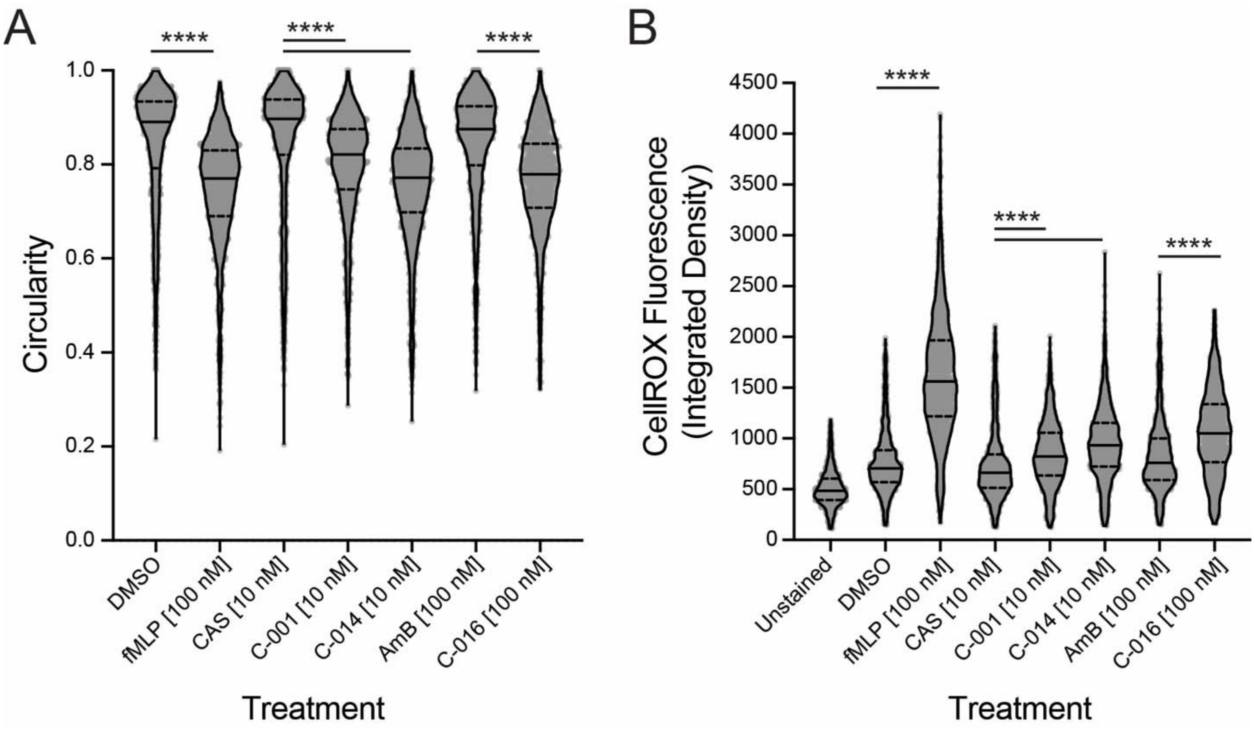

Figure S2. Human neutrophils are activated following treatment with bifunctional compounds. Violin plots show data distribution with mean (solid line) and quartiles (dashed line) with individual points represented by empty grey circles.

A) Following treatment with fMLP [100 nM] or bifunctional compounds, neutrophils exhibited less circularity compared to relevant controls, indicating activation and adhesion of the cells. N $\geq$ 969 cells measured per condition. Statistics: One-way ANOVA, ***p < 0.0001.

B) Staining with the ROS indicator CellROX showed significantly increased ROS production by neutrophils activated with fMLP or bifunctional compounds compared to relevant controls. N $\geq$ 969 cells measured per condition. Statistics: One-way ANOVA, ***p < 0.0001.

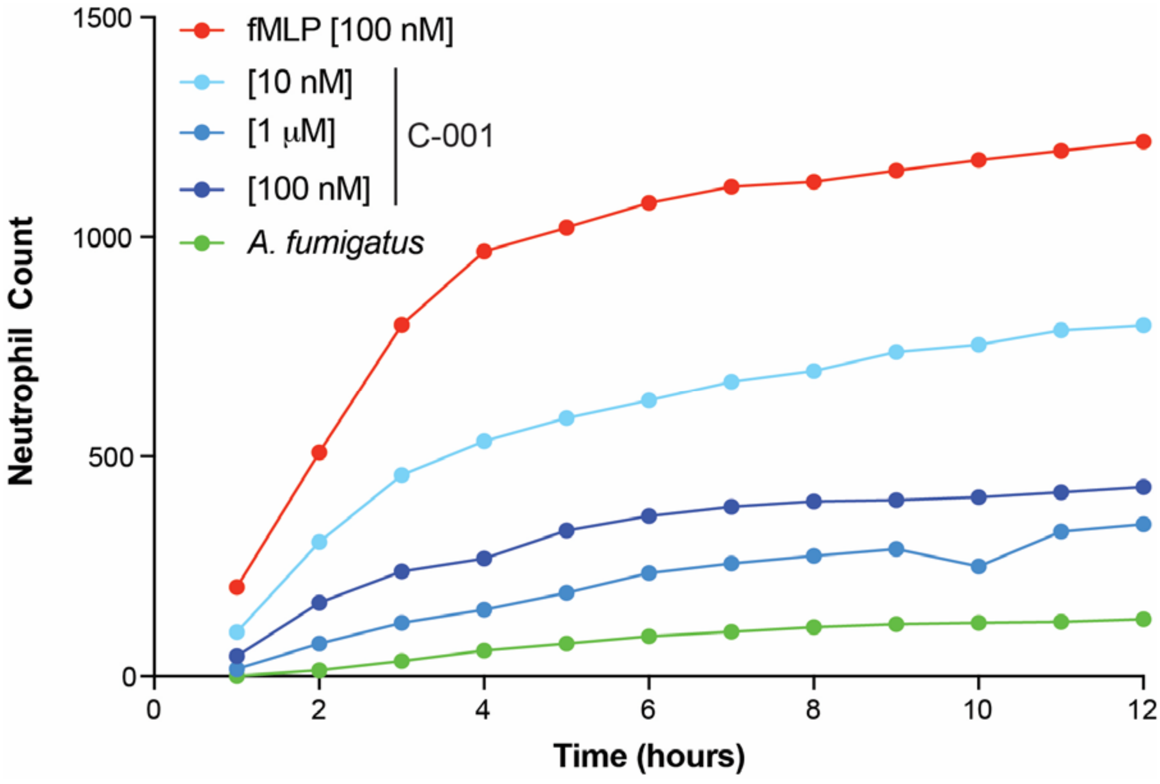

Figure S3. Dose-response curve showing neutrophil recruitment (per device) for C-001 compared to fMLP at [100 nM]. Maximum recruitment to C-001 was observed at [10 nM].

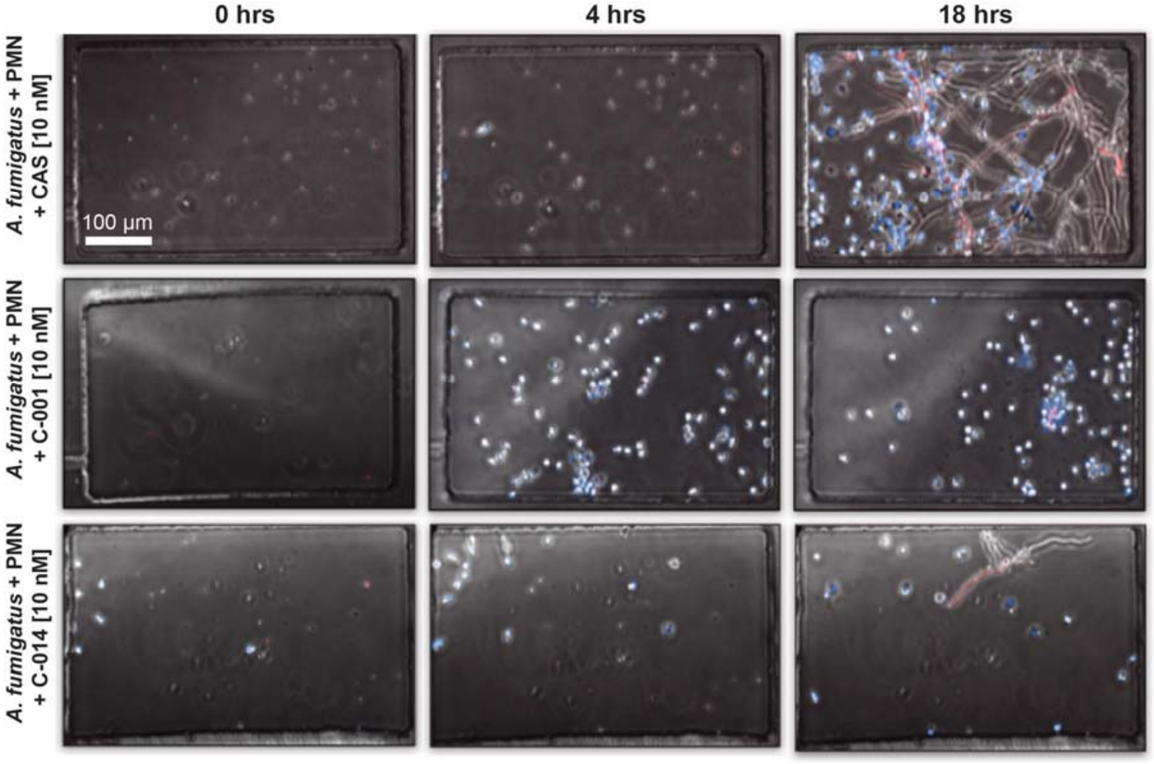

Figure S4. Representative images of chemotaxis chambers containing *A. fumigatus* in the presence of C-001 and C-014 (CAS TM) in comparison to CAS control. Bifunctional compounds recruit neutrophils at earlier time points (4 hrs), which efficiently suppress hyphal growth compared to the CAS alone control.

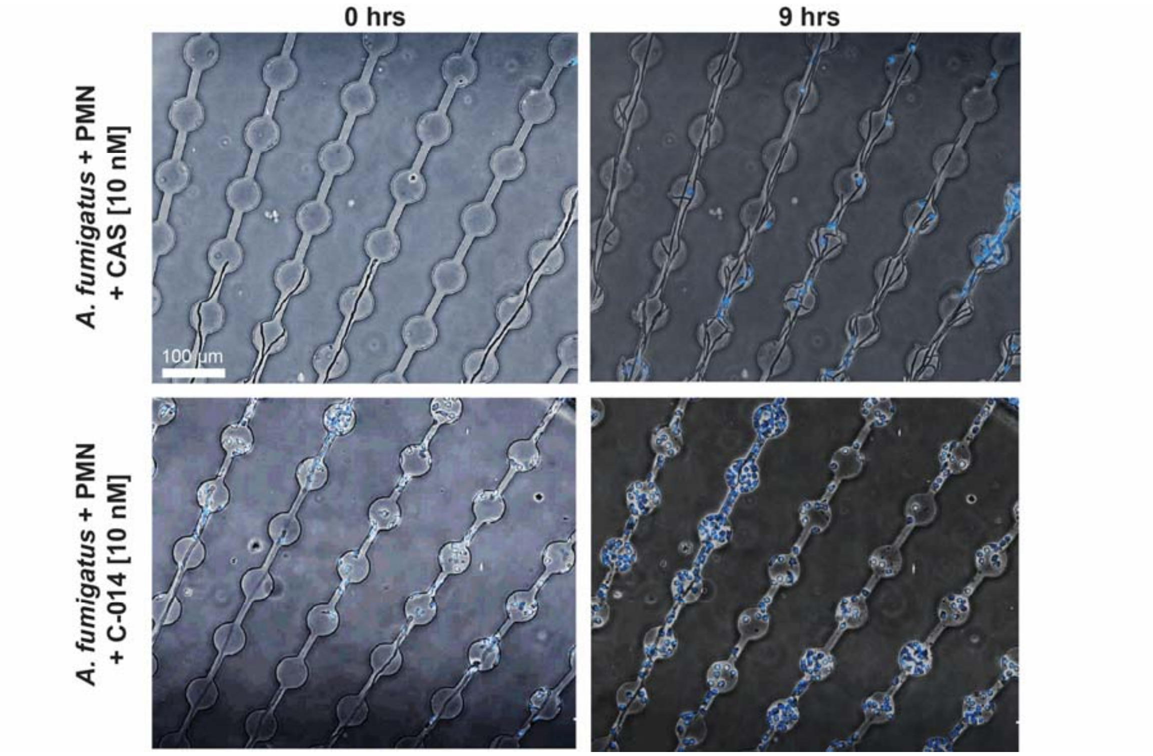

Figure S5. Representative images of neutrophil recruitment towards growing hyphae in the presence of C-014 and CAS control. C-014 increases recruitment of neutrophils to growing hyphae and enhances formation of neutrophil swarms. CAS TM without EM does not enhance neutrophil recruitment to hyphae.

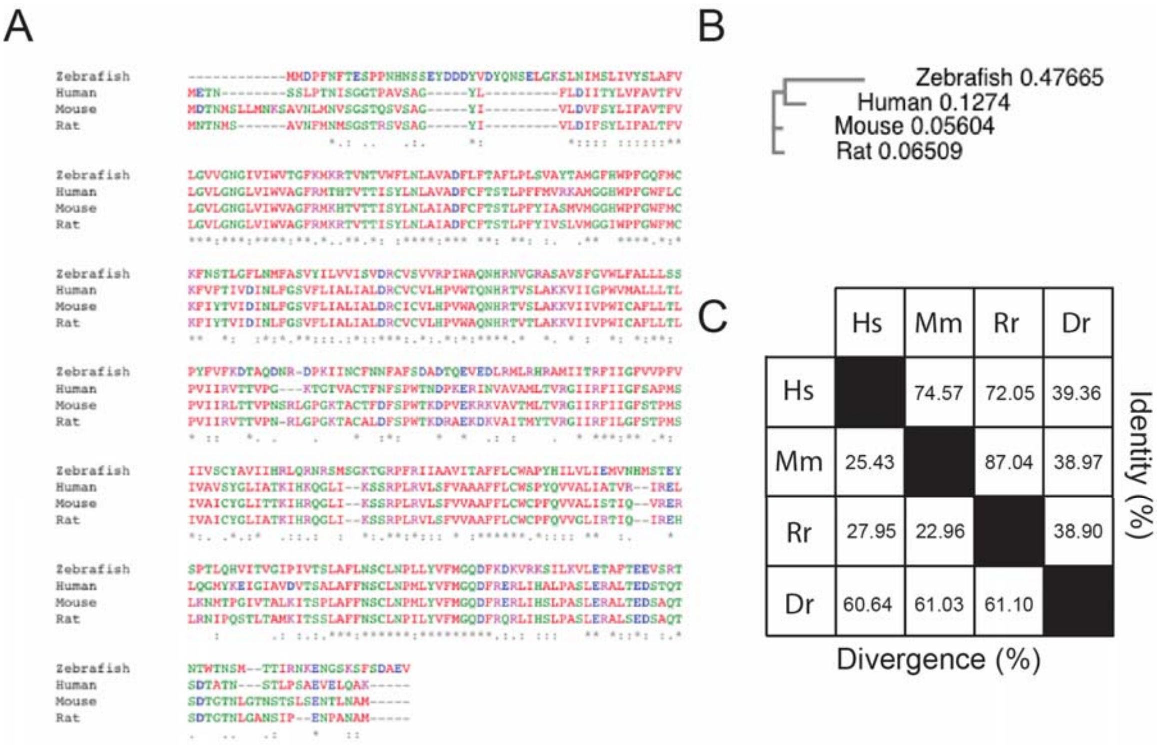

Figure S6. Divergence of formyl-peptide receptor (FPR-1) identity between mammals and zebrafish.

A) Sequence alignment of FPR-1 homologues between species using CLUSTALW2.1.
B) Phylogenetic tree for FPR-1 homologues.
C) Identity matrix of FPR-1 homologues showing percent identity and percent divergence between species.

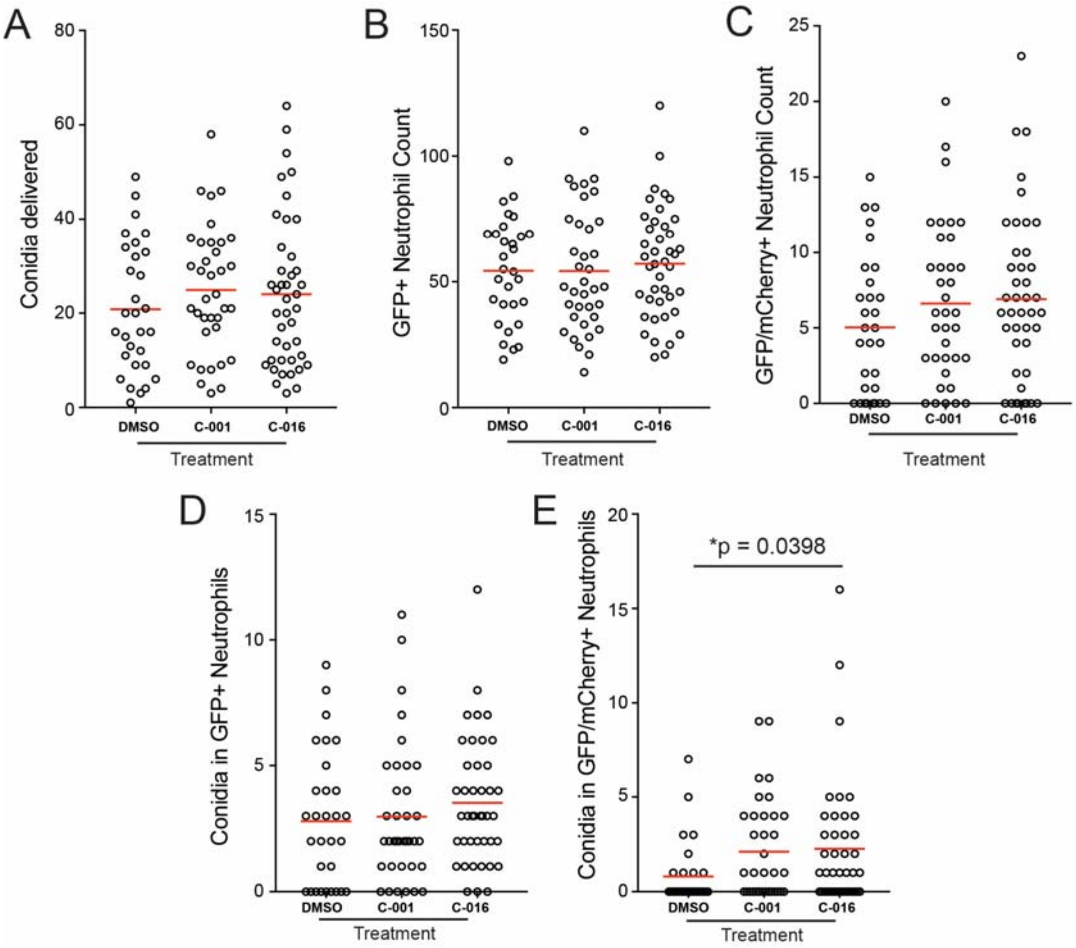

Figure S7. Cell counts and raw phagocytosis scores for zebrafish with mosaic leukocyte expression of human FPR-1.

A) Scatterplot shows equivalent conidial counts per field of view for the different treatment groups.
B) Scatterplot shows equivalent GFP+ cell counts per field of view for the different treatment groups.
C) No significant difference in number of GFP/mCherry+ cells was observed between treatment groups.
D) No significant difference in phagocytosis by GFP(only)+ neutrophils was observed following treatment with bifunctional compounds.
E) Treatment with C-016 resulted in significantly increased phagocytosis of conidia by GFP/mCherry+ (human FPR-1-expressing) neutrophils compared to DMSO-treated controls. N ≥ 40 larva scored per condition. Data collated from N = 2 experiments.

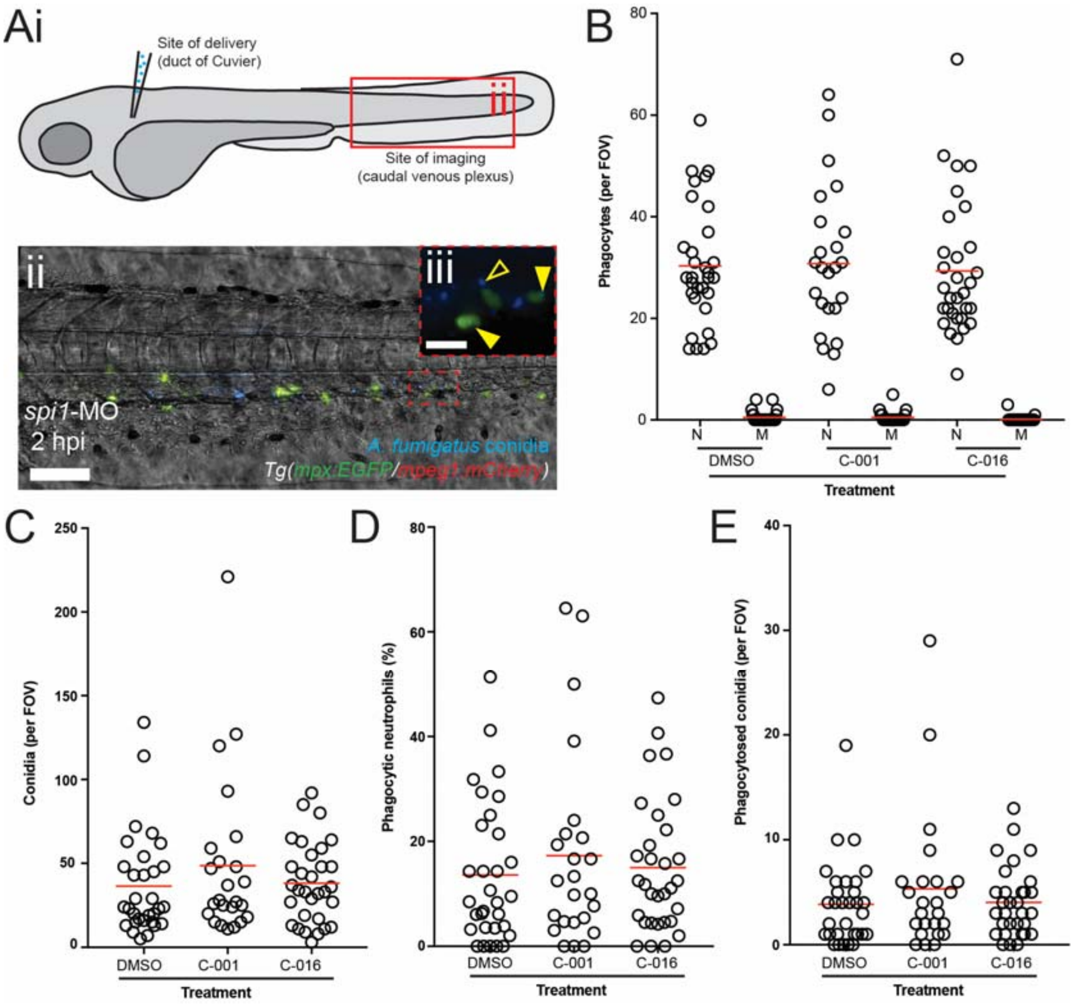

Figure S8. Bifunctional compounds do not enhance phagocytosis of conidia by wild-type zebrafish neutrophils.

A) Diagram of a 72 hpf zebrafish larva indicating the site of inoculation and the site of analysis (i). (ii) Collapsed z-stack (maximum intensity) representative image showing conidia (blue, Hoechst) and neutrophils (green, GFP) in the caudal venous plexus of *Tg(mpx:GFP/mpeg1:mCherry)* embryos injected with *spi1*-MO at the one-cell stage and infected with *A. fumigatus* conidia at 72 hpi. Scale: 100 µm. iii) higher-magnification of neutrophils and conidia indicating extracellular conidia (open yellow arrowhead) and examples of phagocytosis by neutrophils (filled yellow arrowheads). Scale: 20 µm.

B) Graph shows neutrophil (N) and macrophage (M) counts in each caudal venous plexus field of view (FOV) for *spi1*-MO morphant larvae injected with treated and control conidia.

C) Graph shows conidia counts per FOV for each treatment group.

D) Graph shows the percent of neutrophils containing conidia at 2 hpi for each treatment group.

E) Graph shows the number of phagocytosed conidia per FOV at 2 hpi for each treatment group. Each point represents an infected larva.

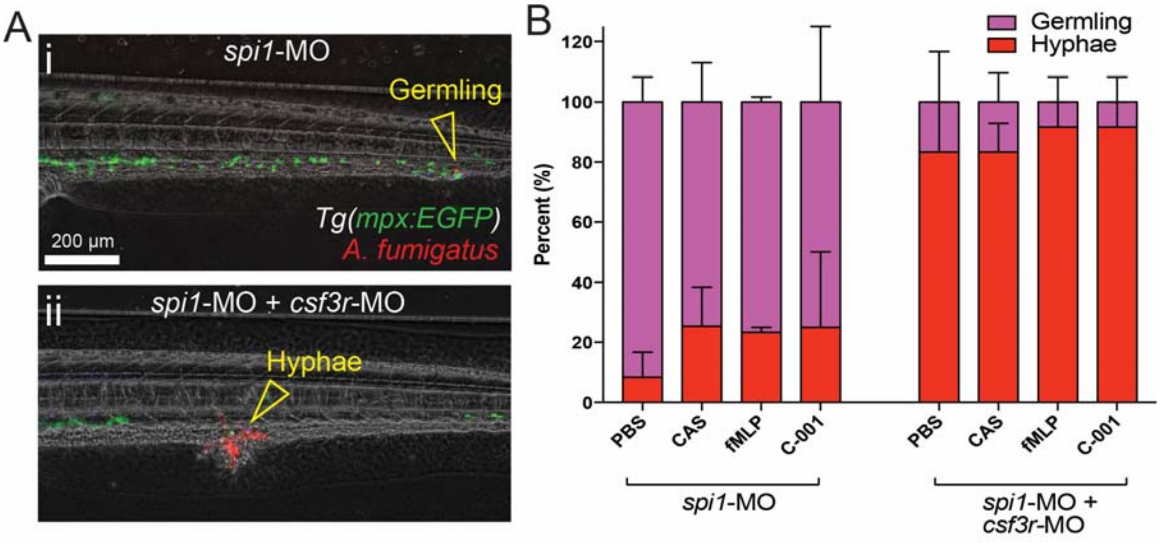

Figure S9: Treatment with bifunctional compounds does not enhance the neutrophil-dependent suppression of fungal hyphae in wild-type zebrafish.

A) Representative images of *spi1* (i) and *spi1/csf3r* (ii) morpholino-injected zebrafish larvae infected with *A. fumigatus* at 1 dpi. Different *A. fumigatus* growth forms are indicated by open yellow arrowheads.

B) Graph showing the proportion of surviving infected embryos with germinating conidia or fully-developed hyphae at 1 dpi. Error bars: Mean + SEM. N = 10 larva per group per experiment, data collated from ≥ 3 experiments.
